# Case Report: Diagnosis of *Klebsiella pneumoniae* Invasive Liver Abscess Syndrome With Purulent Meningitis in a Patient From Pathogen to Lesions

**DOI:** 10.3389/fmed.2021.714916

**Published:** 2021-09-10

**Authors:** Sheng Zeng, Wei-qian Yan, Xiao-mei Wu, Hai-nan Zhang

**Affiliations:** ^1^Department of Geriatrics, The Second Xiangya Hospital, Central South University, Changsha, China; ^2^Department of Neurology, The Second Xiangya Hospital, Central South University, Changsha, China

**Keywords:** *K. pneumoniae* liver abscess syndrome, purulent meningitis, rapid pathogen diagnosis, metagenomic next-generation sequencing (mNGS), case report

## Abstract

As a determinant human pathogen, *Klebsiella pneumoniae* is known to cause rare *K. pneumoniae* liver abscess syndrome (KLAS) which was more common in Asia in early-stage and reported increasingly outside Asia now. Patients with KLAS who have septic metastatic ocular or central nervous system (CNS) lesions are associated with high morbidity and mortality. Relatively infrequent adult community-acquired *K. pneumoniae* meningitis have been documented and most were with poor prognosis. In this paper, we reported a case of KLAS presenting purulent meningitis as disease onset. While negative results were obtained in the bacterial culture of CSF, blood, or liver pus, metagenomic next-generation sequencing (mNGS) of CSF, and blood samples which were synchronously performed demonstrated *Klebsiella pneumoniae* as the pathogenic microorganism (13,470 and 5,318 unique reads, respectively). The ultimately cured patient benefited from rapid pathogen diagnosis, early percutaneous drainage of the abscess, and prompt appropriate antibiotic administration. Our case highlights the importance of clinicians using mNGS for early pathogen diagnosis of this disease.

## Introduction

*K. pneumoniae* is a well-known human nosocomial pathogen although it appears as the normal flora of the human oral cavity and intestine in most situations (1). Most *Klebsiella pneumoniae* community-acquired infections result in pneumonia or urinary tract infections. However, it also causes rare *K. pneumoniae* liver abscess (KLA) in the absence of predisposing factors of hepatobiliary disease (2). KLA was reported most in Asia in early-stage and now it is increasing outside Asia (3, 4). Except for liver abscess, bacteremia dissemination may result in severe complications including endophthalmitis, meningitis, necrotizing fasciitis, and other illnesses. About 13% of patients with KLA have septic metastatic ocular or central nervous system (CNS) lesions which are associated with high morbidity and mortality (5). The rapid diagnosis followed by appropriate treatment may improve the patient's outcome.

Metagenomic next-generation sequencing (mNGS) is becoming a powerful pathogen detection tool with the advantages of high sensitivity, wide-coverage, and high efficiency, which can greatly improve pathogen identification (6). It has now been successfully used for pathogen detection in biological specimens including CSF and blood (7). Early diagnosis assisted by mNGS contributes to timely treatment. In our report, we displayed a case of KLA manifested as purulent meningitis at the onset of the disease. Rapid pathogen diagnosis facilitated further lesion detection, proper treatment, and full recovery of the patient. Until now, few KLA cases with metastatic meningitis were reported, fewer patients achieved a good prognosis (8). Our case suggested the importance of early pathogen diagnosis.

## Case Presentation

A 59-year-old female rural resident in Hunan province, China, was admitted to the Emergency Department (ED) of Second Xiangya Hospital, Central South University. She complained of headache with fever for 6 days and fatigue and somnolence for 1 day. The first symptoms were headache with fever. She experienced four episodes of diarrhea on the first day. No other gastrointestinal symptoms were presented since then. From the second day to the fourth day, she was admitted to a local hospital. A lumbar puncture was performed because of clinical suspicion of “intracranial infection.” The intracranial pressure was 140 mmH_2_O. Cerebrospinal fluid (CSF) was yellow and purulent, and revealed 1,080 white blood cells/μL with a monocyte's ratio of 56.9%. She was immediately treated with intravenous ceftriaxone 2,000 mg q12h. Although the history of diabetes was denied, her random blood glucose was as high as 14 mmol/L during hospitalization. On the fifth day, she was recommended to refer to our hospital due to the new appearance of fatigue and somnolence. Her past medical history showed no recent travel, tick bites, sick contact, alcohol, or drug use. Upon admission, her initial vital signs included a body temperature of 36.7°C, heart rate of 82 beats/min, blood pressure of 125/80 mmHg, respiratory rate of 23 breaths/min, and oxygen saturation of 99% on 2 L/min oxygen. Her consciousness was somnolence. No skin rash was observed, and no obvious abnormality was found on physical examination of the heart, lung, and abdomen. A neurologic examination revealed that the neck was stiff and Kernig's sign was positive. Laboratory test on ED showed a white blood cell count of 9.0 × 10^9^/L with an elevated neutrophil ratio of 83.9% in blood routine test. Liver function test showed hypoalbuminemia (26.1 g/L). There was no significant change in the coagulation panel. The random blood glucose was 15.3 mmol/L. The blood ketone and lactate were 2.43 and 0.9 mmol/L, respectively. Lung computed tomography (CT) scan showed focal small patchy infiltrates in both lower lungs. Considering the severity of the patient's condition, she was sent to the intensive care unit of the department of neurology (NICU) for further treatment. Further laboratory tests and auxiliary examinations were arranged. The concentrations of procalcitonin, C-reactive protein, erythrocyte sedimentation rate (ESR) and interleukin 6 (IL-6) were 8.720 ng/ml, 134.0 mg/L, 45 mm/h, and 630.0 pg/ml, respectively. The result of glycosylated hemoglobin was 10.7%. Thyroid function test results showed that T3 was 0.56mol/L, FT3 was 1.58 mol/L, and T4 was 50.70 mol/L. Two sets of peripheral blood cultures were ordered. Lumbar puncture showed the intracranial pressure was 130 mmH_2_O. Cerebrospinal fluid (CSF) revealed 1,000 white blood cells/μL with proportion of multinucleated cells of 95%, protein of 943 mg/L, and glucose of 3.97 mmol/L (synchronous blood glucose of 14.0 mmol/L). The CSF Gram staining, Ink staining, acid-fast staining, and bacterial culture were all negative. The above findings led to the diagnosis of purulent meningitis, pulmonary infection, diabetes mellitus (DM), and diabetic ketosis. To further identify the pathogen, the PACEseq mNGS test (Hugobiotech, Beijing, China) was also performed on CSF samples on the Nextseq 550 platform (Illumina, San Diego, CA). She has immediately received emergency management of ketosis and was treated with intravenous meropenem 2,000 mg q8h and vancomycin 500 mg q6h on admission (at this moment, the duration of ceftriaxone therapy was 3 days).

**Figure 1:**
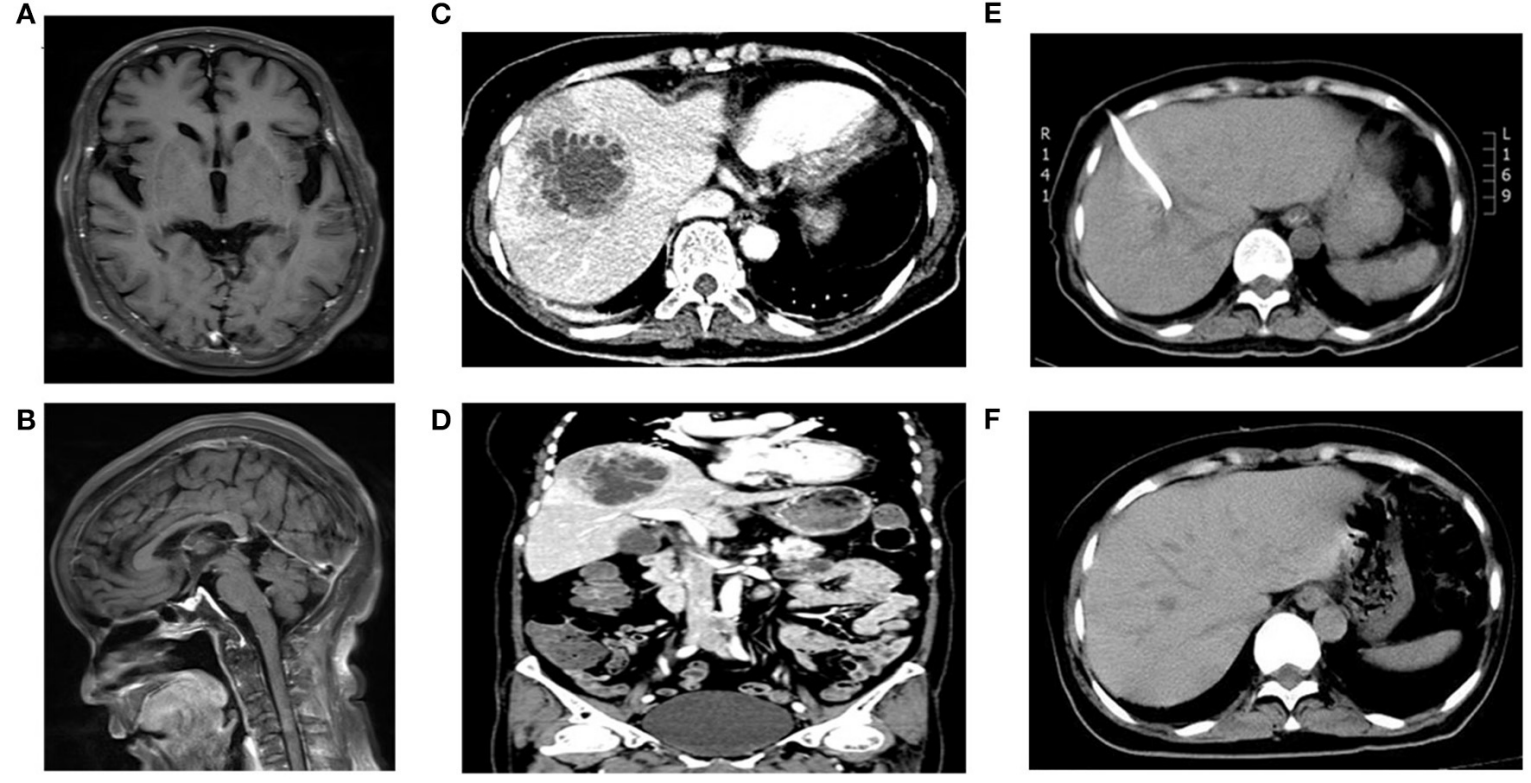
MRI or CT scan images of the patient. **(A,B)** indicated partial enhancement of pia mater in brain enhanced MRI; **(C,D)** revealed an area of abnormal attenuation measuring 67 mm × 62 mm in the right lobe of the liver, indicative of a single large multi-loculated abscess in abdominal enhanced CT; **(E)** displayed the abscess reduced to 51 mm × 37 mm 3 days after emergency CT-guided percutaneous drainage of the liver abscess in abdominal plain CT; **(F)** showed no lesions in liver in the reexamination of abdominal CT after full recovery.

On the second day of hospitalization, a brain MRI scan was performed, and partial enhancement of the pia mater was recommended (Figures 1A,B). She experienced a chill with a body temperature of 38.6°C on this day. Peripheral blood culture was performed. Lumbar puncture and intrathecal injection of 20 mg vancomycin were arranged continuously on the second to the fourth day.

**Figure 2:**
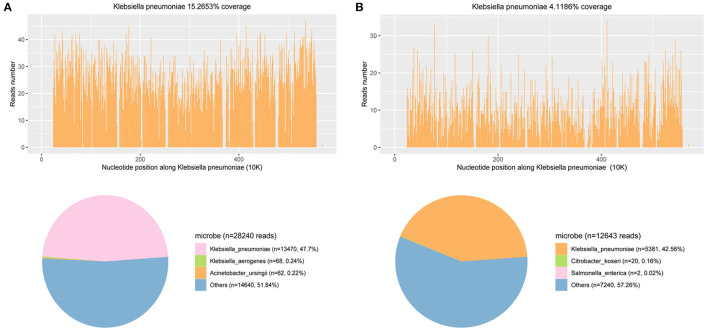
mNGS results of pathogen identification: 47.7 and 42.56% of bacterial reads corresponded to *K. pneumoniae* with coverage of 15.2653 and 4.1186% in CSF **(A)** and blood **(B)**, respectively.

On the fourth day of hospitalization, the mNGS of CSF reported *Klebsiella pneumoniae*, with a total of 13,470 detected unique reads detected (Figure 2A). *Klebsiella pneumoniae* invasive liver abscess syndrome was suspected as the clinical features and unusual findings of the pathogen in this patient. An abdominal enhanced CT demonstrated a single abscess (67 mm × 62 mm) in the right lobe of the liver with separating enhancement (Figures 1C,D) and confirmed our suspicion. No other intra-abdominal pathologies, such as gallstones, were observed. To quickly ascertain the transmission route, mNGS of a blood sample was also arranged. After the diagnosis with *Klebsiella pneumoniae* invasive liver abscess syndrome, the antimicrobials of the patient were adjusted to meropenem 2,000 mg q8h intravenously. An emergency CT-guided percutaneous drainage of the liver abscess was performed, which drained 210 mL of yellow pus over the first 24 h. The liver aspirate was subjected to Gram staining and both aerobic and anaerobic culture. Repeated blood and liver pus cultures were required during episodes of fever.

On the seventh day of hospitalization (the third day after operation), only 20 mL yellow pus was drained over the third 24 h. Reexamination of abdominal CT suggested the liver abscess was reduced to 51 mm × 37 mm (Figure 1E). Lumbar puncture after operation showed the intracranial pressure was 140 mmH_2_O. Cerebrospinal fluid (CSF) revealed 1 white blood cells/μL (mononuclear cells), protein 780 mg/L, and glucose 5.88 mmol/L (synchronous blood glucose of 8.1 mmol/L). Meanwhile, the blood mNGS results demonstrated 5,318 unique reads of *Klebsiella pneumoniae* (Figure 2B). Thus, purulent meningitis with *Klebsiella pneumoniae* was septic metastatic from invasive liver abscess through blood.

On the ninth day of hospitalization (the fourth day after operation), the patient recovered to good condition after treatment. However, the patients and their families strongly requested to return to the local hospital for further treatment. A telephone follow-up after 3 months showed the patient recovered and was discharged from the hospital after 29 days of treatment. Thus, the total duration of treatment was nearly 40 days. The patient underwent abdominal CT again, which showed no lesions in her liver (Figure 1F) at the second follow-up after 9 months.

It should be noted that bacterial cultures of CSF, blood, and liver pus in the whole disease course were all negative.

## Discussion

*Klebsiella pneumoniae* liver abscess syndrome (KLAS) is clinically characterized by bacteremia, liver abscesses, and metastatic infection caused by hypervirulent strains harboring capsular serotype K1 or K2. DM may be a risk factor for this syndrome (2). The most common clinical manifestations of patients with *K pneumoniae* liver abscesses are fever, chills, and abdominal pain. However, all these manifestations lack specificity. For patients, especially those who suffer from diabetes mellitus present with *K pneumoniae* bacteraemia, meningitis, endophthalmitis, or other extrahepatic infections, it is necessary to look for potential liver abscess. Thus, timely identification of pathogens is the key to better management. However, traditional methods for pathogen discovery such as bacterial culture are time-consuming and lack sensitivity (9). The positivity rate of traditional methods is influenced by quality and quantity of specimens, patient antibiotic administration, the severity of infection, and laboratory sufficiency. In our case, the effort was paid through the repeated bacterial cultures of CSF, blood, or liver pus. However, the results were poor and were most likely influenced by empirical antibiotic therapy. The patient's treatment could be delayed without the identification of the pathogen by mNGS.

mNGS emerges as an alternative and efficient molecular diagnostic method, which overcomes the limitations of traditional methods. It is now increasingly applied in the identification of pathogens in clinical practice, such as sepsis, meningitis, and acute respiratory infection (7, 10, 11). The state-of-the-art mNGS technology has advantages in identifying rare, novel, difficult-to-detect, and coinfected pathogens directly from clinical samples, providing timely diagnostic evidence to guide treatment plans (12). In the future, more validated workflows, lower cost, and simplified interpretation criteria would be needed to further routinely implement mNGS in clinical practice. Our patient was an elderly rural woman who had an unrecognized history of DM. She presented with purulent meningitis at the time of onset and was demonstrated as KLAS on the fourth day after admission. After consulting the literature, we found that adult community-acquired *K. pneumoniae* meningitis has been less recorded (8, 13). Unfortunately, few patients have survived this disease. In addition to the severity of the disease, the lack of timely diagnosis and standardized treatment may also be the important reasons.

Until now, there are no clear guidelines for the management of *Klebsiella* pneumoniae invasive liver abscess syndrome with purulent meningitis (2). The basic consensus is the combination of early percutaneous drainage or open (laparoscopic) surgical drainage of the abscess and prompts the appropriate antibiotic administration. The selection of antibiotics is based on *in-vitro* susceptibilities and clinical response. However, results of antibiotics susceptivity are time-consuming. Therefore, the empirical use of high-dose third-generation cephalosporins, including cefotaxime (up to 2,000 mg every 4 h) and ceftriaxone (2,000 mg, twice a day) are options for treatment of *K. pneumoniae* meningitis. Imipenem and meropenem can be given to patients when strains containing extended-spectrum beta-lactamase are suspected. Our patient benefited from the empirically and sufficiently intravenous use of meropenem and early percutaneous drainage of abscesses in our clinic.

In conclusion, we shared a case of KLA that presented with purulent meningitis at the onset of the disease. Rapid pathogen diagnosis through mNGS facilitated further lesion detection, proper treatment, and full recovery of the patient. Nowadays, adult community-acquired *K. pneumoniae* meningitis carries a very poor prognosis. Our case highlights the importance of early pathogen diagnosis by mNGS for the precise treatment of this disease. Meantime, it raises the potential of diagnosing severe infectious diseases from pathogens to lesions.

## Data Availability Statement

The datasets used and/or analyzed during the current study are available from the corresponding author on reasonable request.

## Ethics Statement

Ethical Committee of the Second Xiangya Hospital of the Central South University in China (equivalent to an Institutional Review Board) approved the study, and written informed consent was obtained from the patient for publication of this Case Report and any accompanying images.

## Author Contributions

SZ collected the materials, analyzed the data, and wrote the paper. Wq-Y assisted in clinical follow up. Xm-W and Hn-Z designed the concept and provided administrative support. All authors contributed to the article and approved the submitted version.

## Funding

This work was supported by the Program of National Natural Science Foundation of China (#81801123).

## Conflict of Interest

The authors declare that the research was conducted in the absence of any commercial or financial relationships that could be construed as a potential conflict of interest.

## Publisher's Note

All claims expressed in this article are solely those of the authors and do not necessarily represent those of their affiliated organizations, or those of the publisher, the editors and the reviewers. Any product that may be evaluated in this article, or claim that may be made by its manufacturer, is not guaranteed or endorsed by the publisher.
